# Efficacy of Pharmaceutical Care in Patients with Type 2 Diabetes Mellitus and Hypertension: A Randomized Controlled Trial

**DOI:** 10.1155/2022/7681404

**Published:** 2022-03-24

**Authors:** Weibo Wang, Lijuan Geng, Chenjing Sun, Hui Li, Jinying Wang

**Affiliations:** ^1^Department of Pharmacy, Dongying People's Hospital, Dongying, China; ^2^Department of Pharmacy, The Second People's Hospital of Dongying, Dongying, China; ^3^Department of Quality Management, Dongying People's Hospital, Dongying, China

## Abstract

**Background:**

The rates of treatment adherence and treat-to-target rates of blood pressure and blood glucose-related indexes in patients with hypertension and type 2 diabetes mellitus (T2DM) are associated with prognosis. This study aimed to investigate the efficacy of pharmaceutical care postdischarge for treatment adherence in hypertensive patients with type 2 diabetes mellitus (T2DM).

**Methods:**

This was a randomized controlled trial of patients with combined T2DM and hypertension treated between January and May 2018. Pharmaceutical care included free access to a clinical pharmacist, education material, a WeChat account for live discussion, and a telephone follow-up. The primary endpoint was the 3-month medication adherence. The secondary endpoints included the achieving levels of target rates and values of fasting plasma glucose (FPG), 2 h postprandial glucose (2hPG), and hemoglobin A1c (HbA1c) and the rates of reaching the achieving target rate of blood pressure target <130/80.

**Results:**

In 80 participants, the 3-month medication adherence was higher in the pharmaceutical care group than the routine group (90.0% vs. 52.5%, *P* < 0.001). In terms of FPG, 2hPG, and HbA1c, there were also significant differences between the pharmaceutical care and routine groups (FPG, 6.50 (6.00, 7.18) vs. 7.00 (6.83, 7.78) mmol/L, *P*=0.004; 2hPG, 8.45 (7.45, 9.28) vs. 9.35 (8.23, 10.15), *P*=0.007; HbA1c, 6.5% (6.3%, 7.0%) vs. 7.0% (6.5%, 7.4%), *P*=0.007). The achieving target rate of reaching the blood pressure target in the pharmaceutical care group (92.5%) was significantly higher than that in the routine group (62.5%; *P* < 0.05).

**Conclusion:**

The postdischarge pharmaceutical care program in patients with T2DM and hypertension improves medication adherence.

## 1. What Is Already Known about This Topic?

Untreated hypertension is associated with an increased risk of cardiovascular events and mortality.

The treatment adherence and control rates of patients with hypertension and type 2 diabetes mellitus (T2DM) are low.

## 2. What Does This Article Add?

Three months after discharge, the medication adherence of the pharmaceutical care group was significantly better than that of the routine group.

In terms of FPG, 2hPG, and HbA1c, there were significant differences between the pharmaceutical care and routine groups.

The rate of blood pressure control (92.5%) in the pharmaceutical care group was significantly higher than that (62.5%) in the routine group (*P* < 0.05).

## 3. Introduction

Type 2 diabetes mellitus (T2DM) is a common endocrine disorder characterized by variable degrees of insulin resistance and deficiency, resulting in hyperglycemia. The complications of T2DM include cardiovascular disease, neuropathy, nephropathy, retinopathy, and increased mortality [1, 2]. The worldwide prevalence rates of T2DM were was 9% in men and 7.9% in women in 2014 [3]. Hypertension is a sustained elevation of the systemic arterial blood pressure, commonly defined as systolic blood pressure (SBP) ≥140 mmHg and/or diastolic blood pressure (DBP) ≥90 mmHg [4, 5]. The global prevalence rates of hypertension are 24% in men and 20% in women [6]. Risk factors for hypertension include weight gain and obesity, alcohol use (particularly in men), and hyperinsulinemia [4, 5, 7]. Untreated or uncontrolled hypertension is associated with an increased risk of cardiovascular events and mortality [8]. The pathogeneses of hypertension and T2DM often overlap [9]. About 50% of patients with T2DM also have hypertension; meanwhile, 20%–80% of hypertensive patients suffer from T2DM [10, 11], a rate that can even be as high as 40–80% [11]. The co-occurrence of the two diseases increases mortality due to cardiovascular disease [12], imposing a substantial burden on the patients and healthcare systems.

Hypertension and T2DM are chronic conditions that require lifelong therapy. The treatment of the diseases involves the combined use of many drugs, leading to poor adherence, low treatment and control rates, and high morbidity and mortality [13–15]. A previous study showed a control rate for hypertension of 15% in patients with hypertension and T2DM [13]. Another report revealed that in patients with both conditions, the control rates of hypertension and T2DM were, respectively, 40% and 41% [14]. Although patients have access to comprehensive management by medical workers and medication education by clinical pharmacists, adherence remains poor.

Therefore, the present study analyzed the efficacy of pharmaceutical care after discharge in patients with T2DM and hypertension to improve the therapeutic effect and medication adherence.

## 4. Methods

### 4.1. Ethics Approval

This study was approved by the ethics committee of our hospital (2018-001-02). Written informed consent was obtained from all participants. This study was registered (ChiCTR2000031339).

### 4.2. Study Design and Participants

It was a randomized controlled trial of patients with T2DM and hypertension treated at the Department of Endocrinology of our hospital (Shandong, China) between January and May 2018.

The inclusion criteria were 18–65 years of age, diagnosis of T2DM and hypertension based on the Chinese guidelines for the prevention and treatment of type 2 diabetes mellitus (2017 edition) [16] and the Chinese guidelines for the management of hypertension [17], admitted to the hospital for failing to control the blood glucose or pressure, T2DM and hypertension were well controlled according the evaluation of physicians at discharge, received pharmaceutical care during hospitalization, and receiving antidiabetic and antihypertensive drugs after discharge. The exclusion criteria were the medical history of acute cardiovascular and cerebrovascular diseases and other stress states and pregnant or lactating women. The participants lost to follow-up after discharge were withdrawn.

### 4.3. Randomization and Interventions

The participants were randomized in a 1 : 1 ratio into the routine and pharmaceutical care groups using the random number table method. Pharmaceutical care after discharge was provided by clinical pharmacists to the pharmaceutical care group, while the routine group received routine clinical care only. The doctors prescribed the drugs according to their experience and judgment. During hospitalization, the clinical pharmacists routinely conducted medical advice reviews, drug reorganization, and medication education. The intervention was mainly based on medication education, but unreasonable medical advice audits and drug adjustments could be conducted in rarer cases. The participants were provided with a personalized medication education form at discharge, which mainly included the types of drugs used by the patient, usage and dosage, precautions, adverse reactions, blood glucose control targets, and the treatment methods for hypoglycemia and hypotension. Both groups received clinical pharmacist intervention during hospitalization. Therefore, after discharge, all participants had a similar education level.

The following steps were applied for pharmaceutical care in the intervention group. (1) Set up of a pharmacy clinic. Clinical pharmacists provided consultations every Wednesday and Thursday after the visit with the physician. (2) Design of standardized science education materials. The team produced easy-to-understand texts, comics, audios, videos, and other popular science works to provide individualized medication guidance. Instructions for antidiabetic and antihypertensive drugs were designed and improved, including indications, adverse reactions, precautions, usage and dosage, interactions, storage, and expiration date. Participants' medication time indicator charts were generated. (3) Establishment of a “follow-up service,” WeChat exchange group and WeChat official account. Clinical pharmacists answered questions about drugs raised by participants once a day, provided targeted and individualized guidance, and regularly published related popular science reports. (4) Continuous individualized follow-up. After discharge, the participants were followed by telephone every two weeks. The participants were asked about their drugs, and detailed answers were provided for their needs. They were advised not to change their drugs without authorization and consult doctors and pharmacists in time for useful medications. The intervention lasted 3 months.

The routine group received the following procedure. (1) The participants returned to the hospital every 2 weeks on Wednesdays or Thursdays for regular clinical follow-up, including the prescription of hypoglycemic and antihypertensive drugs and the evaluation of blood sugar, blood pressure, and other indexes. (2) Routine care. Nursing staff followed the participants by telephone once a month and recorded the participants' blood sugar, blood pressure, and medication compliance.

### 4.4. Endpoints

The primary endpoint was the medication adherence rate at 3 months after discharge. Medication adherence was evaluated according to the Morisky-Green test (MGT) [18], including the following four questions. (1) Do you ever forget to take your medicine? (2) Have you ever missed taking your medicine on time? (3) When you feel better, do you sometimes stop taking your medicine? and (4) Have you ever stopped your medicine after feeling that your condition worsened? Scoring “no” to all four questions was scored 4, indicating adherence; “yes” to one, two, three, and four questions were attributed 3, 2, 1, and 0 points, respectively, indicating nonadherence. The evaluation of medication adherence was performed by a pharmacist with 10 years of professional experience, at baseline and 3 months after discharge.

The secondary endpoints included the values of the blood glucose-related indexes and the rates of reaching their targets (fasting plasma glucose (FPG), 2 h postprandial glucose (2hPG), hemoglobin A1c (HbA1c)), and the rates reaching the target of blood pressure at 3 months after discharge. According to the Chinese guideline for the prevention and treatment of type 2 diabetes mellitus (2017 edition) [16], the treatment targets of glucose-related indexes were FPG ≤7 mmol/L and 2hPG <10 mmol/L. The target of blood pressure was <130/80 mmHg [17]. The safety endpoints were the adverse events, included hypoglycemia, rash, and nausea.

### 4.5. Data Collection and Measurements

Participants' baseline features such as age, sex, family history, and chronic complications were recorded. FPG, 2hPG, HbA1c, blood glucose achieving rates of reaching the targets rates, blood pressure achieving target rates, and medication adherence 1 day before discharge were considered baseline data. All the participants were followed after discharge. The outcome measures (including FPG, 2hPG, HbA1c, blood glucose achieving target rate, and blood pressure achieving target rate) were recorded at 3 months after discharge, and medication adherence was reevaluated.

### 4.6. Sample Size

Considering a type I error (*α*) of 0.05, a type II error (*β*) of 0.2, and predicted adherence rates in the two groups of 98% and 78%, respectively (estimated based on previous studies [19–21]), the minimum sample size was calculated as 33 participants/group. Considering the withdrawal of the participants, the sample size was 44 for each group.

### 4.7. Statistical Analysis

Statistical analyses were performed using SPSS 17.0 (SPSS Inc., Chicago, USA). The data were analyzed using the full analysis set (FAS). Categorical data were presented as numbers and percentages (%) and compared by the chi-square test. Continuous data with normal distribution were presented as mean ± standard deviation (SD) and compared by Student's *t*-test. Continuous data with skewed distribution were presented as median (IQR) and compared by the Mann–Whitney *U*-test. Two-sided *P* < 0.05 was considered statistically significant.

## 5. Results

### 5.1. Participants

**Figure 1:**
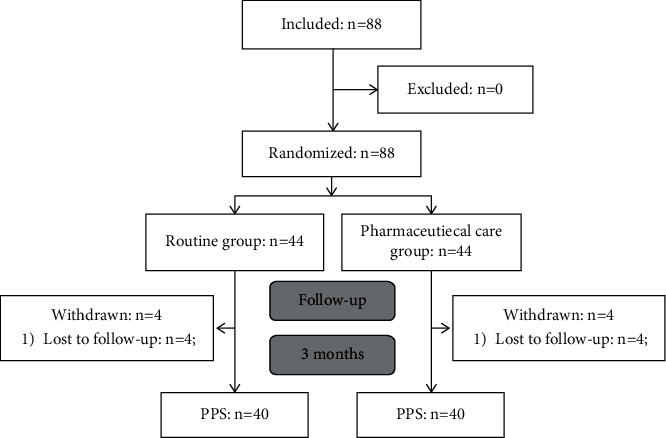
Study flowchart.

A total of 88 participants were enrolled in this study (44/group). After discharge from the hospital, four participants withdrew from each group. Therefore, 40 participants in each group were included in the final analysis (Figure 1).

There were no significant differences in baseline data, including age, sex, BMI, smoking, drinking, family history of T2DM, marital status, chronic complications, chronic diseases, duration of diabetes, and duration of hypertension, between the two groups (Table 1). There were no differences between the two groups in FPG, 2hPG, HbA1c, and blood glucose achieving target rate and blood pressure achieving target rate (all *P* > 0.05) (Table 2).

### 5.2. Efficacy of Pharmaceutical Care at 3 months after Discharge

Three months after discharge, medication adherence was significantly increased in the pharmaceutical care group compared with the routine group (90.0% vs. 52.5%, *P* < 0.001), as given in Table 2. In addition, FPG (6.50 (6.00, 7.18) vs. 7.00 (6.83, 7.78) mmol/L, *P*=0.004), 2hPG (8.45 (7.45, 9.28) vs. 9.35 (8.23, 10.15), *P*=0.007), and HbA1c (6.5% (6.3%, 7.0%) vs. 7.0% (6.5%, 7.4%), *P*=0.007) showed significant differences between the pharmaceutical care and routine groups. There were also significant differences between the two groups in the blood glucose (55.0% vs. 30.0%, *P*=0.032) and blood pressure (92.5% vs. 62.5%, *P* < 0.001) control rates. Finally, HbA1c was higher in the routine group compared with the pharmaceutical care group (7.0% (6.5%, 7.4%) vs. 6.5% (6.3%, 7.0%), *P*=0.007).

### 5.3. Safety of Pharmaceutical Care at 3 months after Discharge

There were five adverse events in the routine group, including two cases of hypoglycemia, two of rash, and one of nausea. Meanwhile, no such event occurred in the pharmaceutical care group (*P*=0.055).

## 6. Discussion

Treatment adherence and achieving target rates in patients with hypertension and T2DM are low [13, 14]. Therefore, this study aimed to investigate the efficacy of pharmaceutical care after discharge in patients with T2DM and hypertension to improve the therapeutic effects and medication adherence. The results indicated that implementing a pharmaceutical care program after discharge in patients with T2DM and hypertension significantly improved medication adherence and the rates of reaching the blood glucose and blood pressure achieving target rates. This study suggested that medication adherence postdischarge was significantly improved after pharmaceutical care compared with routine care. Soto et al. [22] showed that patients with diabetes and hypertension have medication adherence rates ranging from 36% to 93%. Consistent with our findings, pharmaceutical care was shown to improve medication adherence in patients with T2DM and hypertension [23]. In this study, a follow-up service through the WeChat app was established to provide individualized guidance. Patients with medication problems could ask questions at any time and receive answers promptly. Relevant materials of popular science education were also released regularly on the WeChat groups, and the patients were reminded to take medicines on time. Previous evidence suggests that the WeChat platform can provide individualized guidance and improve patient adherence [24]. Still, few studies have assessed medication adherence after discharge in patients with T2DM and hypertension. Pharmaceutical care aims to improve patient awareness of drugs and provide guidance on taking drugs to increase patient adherence. The current findings support the above notion.

The pharmaceutical care group showed no side effects, while the participants who received routine care had a few adverse effects, including hypoglycemia, rashes, and nausea. These findings demonstrated that in addition to efficacy, pharmaceutical care also improves patient safety. Taken together, these data confirm the significance of pharmaceutical care, which should be popularized among hypertensive individuals with diabetes, for improving medicating adherence, drug efficacy, and patient safety. Future studies might further consider factors such as drug types and severity of the condition and more individualized implementation of pharmaceutical care in the real world which could affect the implementation and effectiveness of pharmaceutical care during discharge in order to reduce the burden imposed by these serious ailments on patients and society.

This study had limitations. First, the participants were from a single institution, limiting the generalizability of the current results. In addition, no effort was made to optimize the pharmaceutical care program. The follow-up was short, and there was no assessment of the impact of the intervention and drug adherence on hard outcomes. In addition, the present study was not powered for the assessment of hard outcomes. Patient satisfaction and comments for improvement should be assessed in the future.

## 7. Conclusion

Overall, pharmaceutical care could help patients with T2DM and hypertension after discharge. This care leads to better treat-to-target rates of blood glucose and blood pressure and enhanced medication adherence. Thus, pharmaceutical care has a good clinical application value.

## Figures and Tables

**Table 1:** Baseline characteristics of the patients.

Characteristics	Routine group (*n* = 40)	Pharmaceutical care group (*n* = 40)	*P*
Age (years), median (IQR)	43 (31.0, 50.0)	42 (38.5, 44.8)	0.633
Male, *n* (%)	24 (60.0)	23 (57.5)	0.820
BMI (kg/m^2^), mean ± SD	26.02 ± 3.79	25.85 ± 2.97	0.833
Smoking, *n* (%)	8 (20.0)	10 (25.0)	0.592
Drinking, *n* (%)	5 (12.5)	8 (20.0)	0.363
Family history of DM, *n* (%)	15 (37.5)	17 (42.5)	0.648
Course of DM, *n* (%)			0.688
** **<5 (y)	11 (27.5)	13 (32.5)	
** **5–10 (y)	15 (37.5)	12 (30.0)	
** **>10 (y)	14 (35.0)	15 (37.5)	
Diabetic retinopathy, *n* (%)	10 (25.0)	8 (20.0)	0.592
Diabetic peripheral neuropathy, *n* (%)	10 (25.0)	9 (22.5)	0.793
Diabetic nephropathy, *n* (%)	9 (22.5)	7 (17.5)	0.576
Diabetic foot, *n* (%)	0	0	-
Course of hypertension (years), *n* (%)			0.894
** **<5	3 (7.5)	2 (5)	
** **5–10	20 (50.0)	21 (52.5)	
** **>10	17 (42.5)	17 (42.5)	
Hyperlipidemia, *n* (%)	19 (47.5)	21 (52.5)	0.655
Arteriosclerosis, *n* (%)	18 (45.0)	16 (40.0)	0.581
Coronary heart disease, *n* (%)	12 (30.0)	10 (25.0)	0.617
Hyperuricemia, *n* (%)	4 (10.0)	5 (12.5)	>0.999

IQR, interquartile range; BMI, body mass index; DM, diabetes mellitus.

**Table 2:** Observation indexes at baseline and at 3 months.

Characteristic	Routine group (*n* = 40)	Pharmaceutical care group (*n* = 40)	*P*
Baseline			
** **FPG (mmol/L), median (IQR)	6.30 (6.00, 6.64)	6.40 (6.00, 7.00)	0.497
** **2hPG (mmol/L), median (IQR)	8.85 (8.20, 9.20)	8.50 (8.05, 9.00)	0.422
** **HbA1c (%), median (IQR)	7.80 (6.63, 8.00)	7.10 (6.43, 7.80)	0.072
** **Blood glucose achieving target rate, *n* (%)	37 (92.5)	32 (80.0)	0.095
** **Blood pressure achieving target rate, *n* (%)	40 (100.0)	40 (100.0)	>0.999
** **Medication adherence rate, *n* (%)	38 (95.0)	39 (97.5)	0.556
At 3 months			
** **FPG (mmol/L), median (IQR)	7.00 (6.83, 7.78)	6.50 (6.00, 7.18)	0.004
** **2hPG (mmol/L), median (IQR)	9.35 (8.23, 10.15)	8.45 (7.45, 9.28)	0.007
** **HbA1c (%), median (IQR)	6.95 (6.50, 7.38)	6.45 (6.30, 7.00)	0.007
** **Blood glucose achieving target rate, *n* (%)	12 (30.0)	22 (55.0)	0.032
** **Blood pressure achieving target rate, *n* (%)	25 (62.5)	37 (92.5)	0.001
** **Medication adherence rate (primary endpoint), *n* (%)	21 (52.5)	36 (90.0)	<0.001

IQR, interquartile range; FPG, fasting plasma glucose; 2hPG, 2 -h postprandial glucose; HbA1c, glycated hemoglobin.

## Data Availability

The data used to support the findings of this study are available from the corresponding author upon request.

## Supplementary Materials

Supplementary Materials Supplementary 1. CONSORT checklist.
